# A Prospective Method for the Dynamic Transformation of Structural Balance in Fully Signed Networks

**DOI:** 10.3390/e28010085

**Published:** 2026-01-11

**Authors:** Zhanyong Jiao, Jiarui Fan, Ruochen Zhang, Dinghan Duan

**Affiliations:** 1 School of Marxism, Xi’an Jiaotong University, Xi’an 710049, China; jiao2012078@mail.xjtu.edu.cn; 2 School of Economics and Management, Xi’an University, Xi’an 710049, China; fanjiarui@stu.xjtu.edu.cn; 3 School of Economics and Management, Xi’an Shiyou University, Xi’an 710312, China; 4 School of Economics, Jiangxi University of Finance and Economics, Nanchang 330013, China; 15909130112@163.com

**Keywords:** signed networks, structural balance theory, network optimization, memetic algorithm, computational social science

## Abstract

Structural balance in fully signed networks, integrating both individual attributes and relationships, represents a critical challenge in social computing; however, its dynamic transformation remains underexplored. This study extends structural balance theory by incorporating node attributes and formulating a mathematical framework for optimizing balance dynamics in fully signed networks. A memetic algorithm is designed to achieve structural balance with minimal cost. Evaluations on both synthetic and real-world networks demonstrate the proposed method’s effectiveness, efficiency, and social interpretability.

## 1. Introduction

Social networks commonly exhibit a mixture of affiliative, trusting, or cooperative interactions (positive relationships) alongside those characterized by conflict, disagreement, or competition (negative relationships) [1]. Networks simultaneously containing positive and negative ties are known as signed networks and have been widely investigated across computer science, biology, economics, and political science [2,3,4,5,6,7].

Structural balance theory provides a foundational framework for understanding the evolution of signed networks [8]. Originating from psychological theory, Heider [9] proposed that a signed network is balanced when every triad contains either three positive ties or exactly one positive tie. Cartwright and Harary [10] later formalized this concept using graph theory, demonstrating that a balanced network can be partitioned into two mutually antagonistic clusters or a single cohesive group. Davis [11] subsequently introduced weak structural balance, allowing networks to split into more than two clusters by treating triads with three negative edges as balanced.

Over the past decades, numerous mathematical models have been developed to analyze structural balance and its detection [12,13,14,15,16,17]. These studies provide valuable insights for social science applications, including political polarization, alliance stability, and cooperation–conflict dynamics in organizations. Despite these advances, conventional structural balance models rely primarily on adjacency information and overlook node-level attributes, limiting their explanatory power in social computing. Empirical systems frequently exhibit attribute-driven behavioral heterogeneity. For example, in international relations, ideological similarity may shape cooperation beyond formal alliances; in organizational innovation diffusion, adoption often depends on node attributes such as capacity and risk tolerance; in epidemic networks, individual health behaviors strongly influence transmission outcomes. Ignoring such attributes leads to oversimplified models that fail to capture real-world complexity.

Du et al. [18] extended structural balance to fully signed networks by assigning signs to both nodes and edges, while this provides a vital foundation, four key limitations persist in the existing studies. First, most models are restricted to binary attributes, failing to capture the multi-category nature of social groups. Second, they often assume strong balance, whereas empirical networks frequently exhibit weakly balanced, multi-factional structures [17]. Third, the focus remains on balance detection rather than the dynamic transformation process. Finally, prior models rarely account for the asymmetry in transformation costs, where the social or cognitive effort required to modify a node attribute typically differs from that of an edge sign. Our study addresses these gaps by proposing a unified optimization framework that incorporates multi-category attributes, weak balance, and asymmetric costs.

While various memetic-algorithm-based methods have been proposed for structural balance optimization, they often encounter limitations when applied to fully signed networks. A common challenge is the tendency to be trapped in local optima due to the vast and complex search space of balance dynamics. To bridge this gap, this study develops an enhanced memetic algorithm that distinguishes itself in two key aspects. First, we introduce a synergistic optimization strategy that couples a global genetic search with a fine-grained local search operator, effectively mitigating premature convergence and ensuring the discovery of higher-quality solutions. Second, our method is specifically optimized for the dynamic transformation of balance, providing a more comprehensive and efficient framework than traditional methods.

The primary contribution of this study lies in addressing the dynamic transformation process within fully signed networks, a critical yet overlooked dimension in structural balance research. Unlike traditional approaches focusing exclusively on edge sign adjustments, our framework recognizes that in real-world social systems, individual attributes and interpersonal relationships are intrinsically coupled and co-evolve. By formulating this as a joint optimization problem, we capture the essence of genuine human behavior—where individuals may alter both their internal states (attributes) and external connections to alleviate structural tension.

This work extends structural balance theory from static categorization to a dynamic, multi-dimensional evolutionary process. From the perspective of Computational Social Science, this integrated approach provides a sophisticated methodology for analyzing complex social phenomena, such as opinion polarization and group segregation, where the interplay between “who people are” (nodes) and “how they interact” (edges) is fundamental. Thus, the novelty of this work resides in the synthesis of these components into a unified dynamic model that offers both theoretical depth and practical applicability to evolving social structures.

## 2. Structural Balance and Its Dynamic Transformation

**Figure 1:**
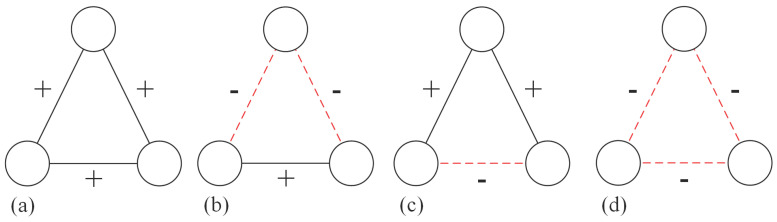
All possible triangle configurations in the general signed network. Solid lines marked with “+” indicate positive links, whereas dashed lines with “−” denote negative relations. Under Heider’s structural balance theory, configurations (**a**,**b**) are classified as balanced, while those in (**c**,**d**) are considered unbalanced. However, (**d**) is considered balanced according to Davis’ theory (weak structural balance).

Heider [9] initially introduced the concept of structural balance using the triad as the fundamental unit, classifying triangles into four distinct types (Figure 1). A network is considered balanced when all its triangles conform to types (a) or (b), adhering to the principles that “a friend of a friend is a friend” and “an enemy of an enemy is a friend.” However, this criterion is often considered overly restrictive for empirical systems. Davis [11] subsequently expanded this notion to “weak structural balance,” wherein type (d) is also deemed balanced. This extension incorporates the logic that “menemies of enemies may remain enemies.”

**Figure 2:**
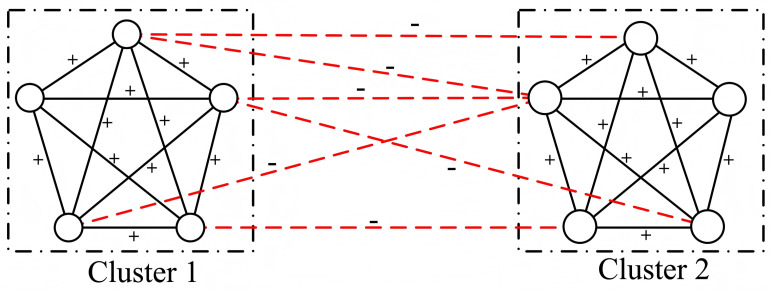
Illustration of a balanced network structure from the global view. Solid lines denote positive connections, and dashed lines denote negative relations. According to the theory proposed by Cartwright and Harary, the network can be partitioned to two opposing clusters of friends, i.e., all edges within Cluster 1 and Cluster 2 are “+”, and those edges between the two clusters are “−”.

Drawing on graph theory, Cartwright and Harary [10] demonstrated that nodes in a strongly balanced signed network can be partitioned into two mutually antagonistic clusters. Such a network is balanced if all node pairs within the same cluster are linked by positive edges, while all inter-cluster pairs are connected by negative edges (Figure 2). Essentially, Cartwright and Harary’s formulation formalized Heider’s original conception. A weaker condition permits the division of the network into multiple clusters to accommodate more complex social structures.

In social computing, Du et al. [18] extended structural balance to “fully signed networks,” where both nodes and edges are assigned “+” or “−” signs. It should be noted that the interpretations of these signs differ: for nodes, “+” and “−” indicate binary attributes such as political party (blue or red), whereas for edges, they represent positive or negative relationships. A signed network can be represented as a graph G=(V,E), where the set of nodes $v_{i}\in V$ is mapped to {+1,−1}, and the set of edges $e_{ij}\in E$ is mapped to {+1,−1}.

**Figure 3:**
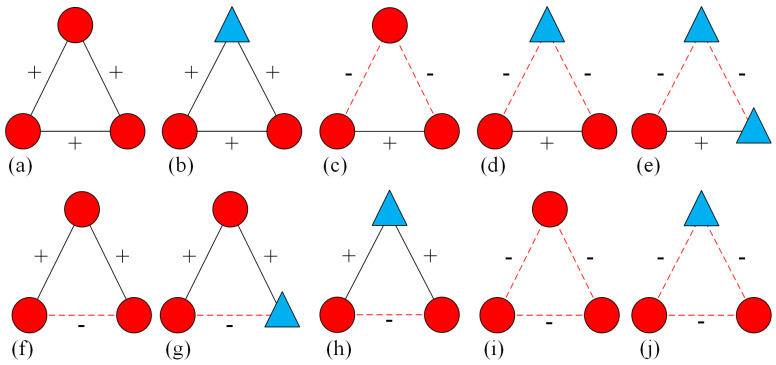
All possible triangle configurations in the fully signed network. Solid lines marked with “+” indicate positive links, whereas dashed lines with “−” denote negative relations. Red circles represent nodes with attribute “+”, while blue triangles represent nodes with attribute “−”. According to the balance theory proposed by Du et al. [18], only configurations (**a**–**d**) are classified as balanced, while configurations (**e**–**j**) are considered unbalanced due to the presence of conflicting node-edge relations.

The various tripartite relationships involving node attributes are shown in Figure 3. Based on the mechanisms of homophily and xenophobia [19], Du et al. [18] postulated that positive edges should connect nodes sharing identical attributes, while negative edges should link nodes with dissimilar attributes; consequently, only triangles (a) and (d) are balanced.

**Figure 4:**
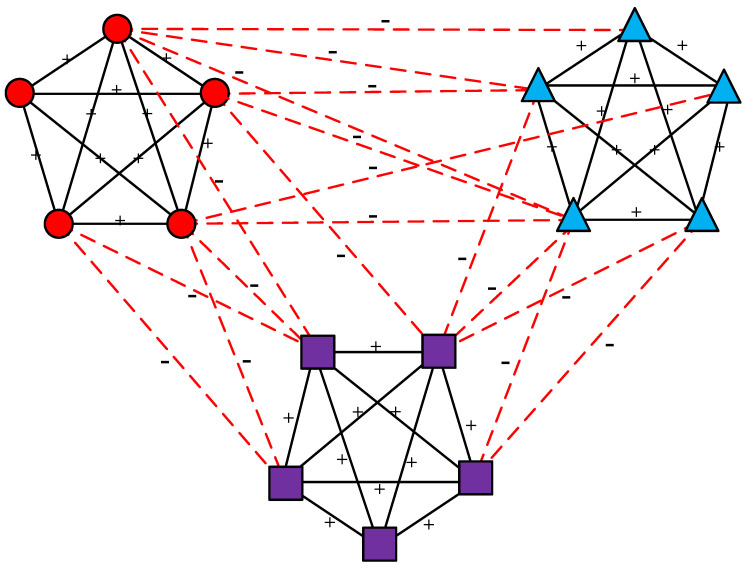
Structural balance in fully signed networks with multi-category attributes. Edges marked with a “+” correspond to positive relationships, depicted as solid lines, whereas those labeled with a “−” indicate negative relationships and are shown as dashed lines. Node attributes are distinguished by varying shapes and colors, where each distinct shape/color combination represents a specific attribute category. For example, there are three attribute categories in the figure: red circle, blue triangle, and purple square.

A primary limitation of this framework is its restriction to binary node attributes. In real-world platforms like Twitter or Reddit, individual attributes are multi-faceted. For instance, political orientation is rarely a simple binary of “left” or “right,” but rather a spectrum including progressive, liberal, centrist, conservative, and libertarian views. Similarly, primary interests can span technology, sports, gaming, or research. Furthermore, the original framework only supports “strong structural balance,” which He et al. [17] argued is too rigid to accurately model real systems. To fill this gap, the binary attribute representation must be extended to a multi-category schema, where the node set is redefined as $v_{i}\in V\to \left\{1,2,3,\ldots \right\}$, with each integer representing a distinct attribute type. When extending this theory to the weak structural balance condition, the network can be globally partitioned into multiple clusters, as illustrated in Figure 4. Within each cluster, nodes share identical attributes and are interconnected by positive edges. In contrast, nodes belonging to different clusters possess distinct attributes and are linked by negative edges.

The triangle theory of structural balance interprets social behaviors from a micro-level perspective. The transition from imbalance to balance represents an inherent trend in the evolution of human interactions [20]. To mitigate conflicts arising from unbalanced relationships, each agent continuously adjusts its interpersonal connections until all its local triangles satisfy balanced conditions. Enhancing the efficiency of structural balance evolution can significantly improve group cohesion and facilitate knowledge dissemination. In real-world systems, however, modifying relationship attributes and node attributes incurs certain costs. For instance, in organizational settings, altering interpersonal relationships may require conflict mediation or trust-building activities, while changing node attributes, such as shifting a person’s stance in a debate, could involve persuasive communication or training. This paper focuses on achieving network balance with minimal cost under such constraints.

**Figure 5:**
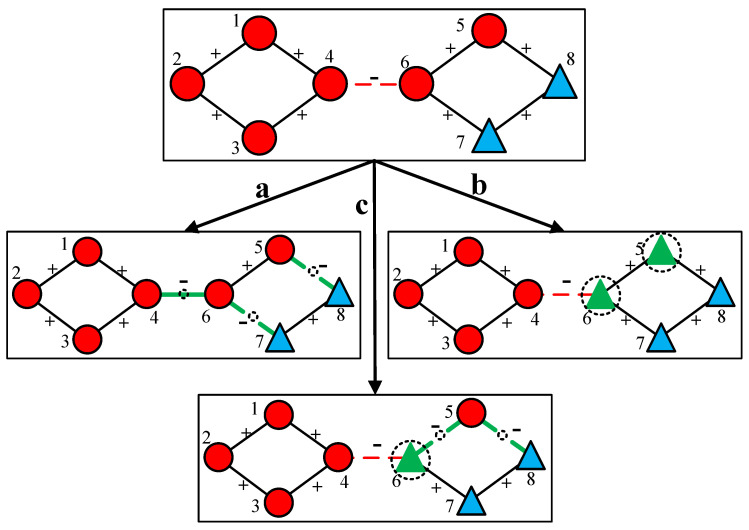
Transformation of structural balance in fully signed networks with multi-category attributes. The upper panel shows the initial unbalanced network, while (a–c) illustrate three distinct pathways to achieve balance: (a) modifying only edge relations (between nodes 4–6, 5–8, and 6–7); (b) altering only node attributes (nodes 5 and 6); (c) a hybrid approach adjusting both edges (5–6, 5–8) and node attributes (node 6). Edges marked with “+” are solid lines, and “−” are dashed lines. The bold green lines and green triangles highlight the specific elements undergoing transformation. The dashed circles are used to group nodes and edges to visually distinguish the different transformation pathways and their affected local structures.

In practice, transforming structural balance can be achieved by multiple ways. As illustrated in Figure 5 the upper panel depicts an initial unbalanced network. Three distinct pathways are presented to transform this network into the balanced one. Path *a* involves modifying three edges: edges between nodes 4 and 6, 5 and 8, and 6 and 7. Path *b* alters the attributes of two nodes, namely node 5 and node 6. Path *c* adjusts both relational and nodal properties by changing edges between 5 and 6, 5 and 8, along with the attribute of node 6. When the costs associated with modifying node edge attributes differ, different appropriate strategies can be selected to optimize the efficiency of structural balance attainment.

To determine the minimal set of nodes or edges whose attributes need to be transformed during the dynamical evolution of signed networks, a suitable metric is required to evaluate whether the network has reached a balanced state. Various indices have been introduced to quantify structural balance, such as the ratio of balanced loops to all loops. However, these conventional approaches suffer from high computational complexity due to the enumeration of cycles. Alternatively, Facchetti et al. [21] proposed an energy function that substantially reduces computational overhead. The energy function is defined as Equation (1), where $e_{ij}$ denotes the sign of the edge between nodes *i* and *j*, and $s_{i},s_{j}\in \left\{+1,-1\right\}$ indicate the cluster assignments.

(1) $$H\left(s\right)=\sum_{i,j}\frac{\left(1-e_{i,j}\cdot s_{i}\cdot s_{j}\right)}{2}$$

In extending this energy function to fully signed networks, the cluster assignment variable *s* was replaced by node attribute *n* in the work of Du et al. [18]. To adapt the function for weak structural balance, a modified version is introduced here, as formalized as Equation (2). In this expression, $n_{i}$ and $n_{j}$ belonging to {1,2,3,…} indicate the attributes of nodes *i* and *j*. The term ⊕ indicates whether both nodes share the same attribute; specifically, $n_{i}⊕n_{j}=1$ if nodes *i* and *j* share the same attribute, and $n_{i}⊕n_{j}=-1$ otherwise. The minimal value of this function corresponds to the number of imbalanced node pairs. An energy value of zero indicates a fully balanced signed network, while higher values reflect greater degrees of imbalance.

(2) $$E=\sum_{i,j}\frac{\left(1-e_{i,j}\cdot n_{i}⊕n_{j}\right)}{2}$$

Building upon this foundation, we formulate the objective function as minimizing the cost required to achieve structural balance throughout the network. A tunable parameter δ belonging to [0,1] is introduced to reflect the fact that modifying edge and node attributes may incur different costs and probabilities in real systems. By adjusting δ, the relative impact of edge modifications versus node updates can be controlled, influencing both the total number and the type of elements altered. The optimization problem for the dynamical evolution toward structural balance is formally defined as

(3) $$\min F\left(x\right)=\delta \cdot x_{edge}+\left(1-\delta \right)\cdot x_{node}+\beta \cdot E$$

where δ represents the cost of transforming edges, while 1−δ represents the cost of transforming nodes. $x_{edge}$ and $x_{node}$, respectively, denote the number of edges and nodes that are transformed, *E* remains consistent with Equation (2), and β is the punishment coefficient to ensure that the energy function reaches 0.

## 3. Algorithm for Optimizing Structural Balance in Fully Signed Networks

### 3.1. Framework and Algorithm

As noted by Facchetti et al. [21], determining structural balance with the lowest energy function is equivalent to finding the ground state of an Ising spin glass model, which constitutes a NP-hard problem. Consequently, conventional algorithms often struggle to solve it efficiently. The memetic algorithm (MA), which emulates cultural evolution by combining genetic algorithms with heuristic local search, has been developed to improve the quality of the solution [22]. The algorithm guides a population from a highly diverse initial state to a near-optimal solution [23]. MA has gained significant attention in computer science and has proven more efficient and effective than standard genetic algorithms in certain applications [24]. They have also been applied to optimize problems in social networks; for instance, a series of studies utilize MAs for structural balance detection, optimizing small-world property, or network sampling [17,25,26]. In this study, we extend the MA framework to develop a novel approach for optimizing the dynamic transformation of structural balance in fully signed networks.

The framework of the proposed method is described in Algorithm 1. The process begins by inputting the network adjacency matrix, node attributes, and a set of key parameters. An initial population PI is generated via the Initialize-Population(·) function (the detailed initialization procedure is presented in Algorithm 2). The iterative process continues until a termination condition is met, i.e., reaching a maximum number of iterations (1000 iterations in this paper) or observing no improvement in the energy function and objective function over a specified interval (100 iterations in this paper). During each iteration, a parent population $P_{\mathrm{parent}}$ is selected through tournament selection. Genetic operations including crossover and mutation are then applied to produce an offspring population $P_{\mathrm{offspring}}$. Subsequently, a local search procedure refines the offspring, yielding $P_{\mathrm{offspringNew}}$. The population is updated by integrating promising solutions from the current population, parent set, and improved offspring. Finally, the best solution is decoded from the final population, indicating the specific transformation strategy.
**Algorithm 1** Framework of the proposed algorithm**Require:** 
PopSize (Population size), TournSize (Tournament size), PoolSize (Mating pool size), *N* (Network size), NumAtt (Number of node attributes), $P_{c}$ (Crossover probability), $P_{m}$ (Mutation probability), δ (Cost of edge’s transformation), *A* (Network adjacency matrix)1:Initialize population PI←Initialize-Population(PopSize,N,NumAtt,δ,A,NA)2:**repeat**3:    $P_{\mathrm{parent}}\leftarrow \mathrm{Tournament}\text{-}\mathrm{Selection}\left(PI,\mathrm{TournSize},\mathrm{PoolSize}\right)$4:    $P_{\mathrm{offspring}}\leftarrow \mathrm{Genetic}\text{-}\mathrm{Operation}\left(P_{\mathrm{parent}},P_{c},P_{m},N,\delta \right)$5:    $P_{\mathrm{offspringNew}}\leftarrow \mathrm{Local}\text{-}\mathrm{Search}\left(P_{\mathrm{offspring}},N,\mathrm{NumAtt}\right)$6:    $P\leftarrow \mathrm{Update}(P,P_{\mathrm{parent}},P_{\mathrm{offspringNew}})$7:**until** termination condition is met8:Decode the best solution from *P*9:**Output the fittest solution**

**Algorithm 2** Initialization operation
**Require:** 
PopSize (Population size), NumAtt (Number of node attributes), δ (Cost of edge’s transformation), *A* (Network adjacency matrix), NA (Node attributes), Compute number of positive edges $N_{\mathrm{pos}}$ and number of negative edges $N_{\mathrm{neg}}$ based on *A*  1:Initialize an empty population set P←[]  2:Set m←1  3:**while** m≤PopSize **do**  4:    Create an empty chromosome C←[]  5:    **for** i=1 to $N_{\mathrm{pos}}+N_{\mathrm{neg}}$ **do**  6:          Randomly create a gene $g_{i}\in \left\{+1,-1\right\}$  7:          Append $g_{i}$ to chromosome *C*, i.e., $C\leftarrow \mathrm{merge}(C,g_{i}))$  8:    **end for**  9:    **for** $i=N_{\mathrm{pos}}+N_{\mathrm{neg}}+1$ to $N_{\mathrm{pos}}+N_{\mathrm{neg}}+N$ **do**10:          Randomly create a gene $g_{i}\in \left\{1,2,\ldots ,\mathrm{NumAtt}\right\}$11:          Append $g_{i}$ to chromosome *C*12:    **end for**13:    **for** $i=N_{\mathrm{pos}}+N_{\mathrm{neg}}+1$ to $N_{\mathrm{pos}}+N_{\mathrm{neg}}+N$ **do**14:          Let *r* be a random value in [0,1]15:          **if** r≥δ **then**16:            Set $g_{i}=NA_{i-N_{\mathrm{pos}}-N_{\mathrm{neg}}}$17:          **else**18:            Randomly choose a positive adjacent node *l* of $i-N_{\mathrm{pos}}-N_{\mathrm{neg}}$19:            Set the gene of *l* to *i*, $g_{i}=g_{l}$20:          **end if**21:    **end for**22:    Add *C* to population *P*, i.e., P←merge(P,C)23:    Increment m←m+124:
**end while**
25:**Output:** Population *P*


**Figure 6:**
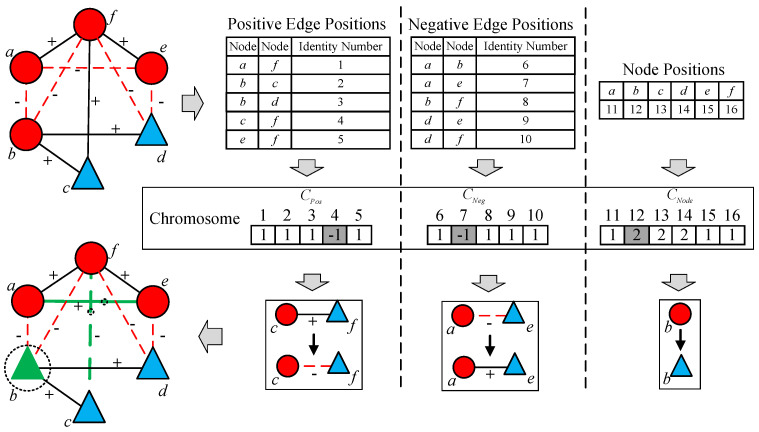
An illustration of representation. An initial network is shown in the upper left, where edges marked with “+” are solid lines and “−” are dashed lines; node attributes are distinguished by varying color/shapes (details refer to Figure 4). The chromosome maps specific genes to network positions: in strings $C_{\mathrm{Pos}}$ and $C_{\mathrm{Neg}}$, genes 4 and 7 are assigned −1, indicating that the signs of edges between nodes *c*–*f* and *a*–*e* are swapped (bold green lines). In string $C_{\mathrm{Node}}$, gene 12 differs from the initial attribute, meaning node *b*’s attribute is transformed from 1 (red circle) to 2 (green triangle). The gray background in the chromosome is used to visually distinguish the segments for edge transformation and node attribute transformation.

We employ a comprehensive encoding scheme for both edges and nodes. Each solution is represented by a composite string: $C=\{C_{\mathrm{Pos}},C_{\mathrm{Neg}},C_{\mathrm{Node}}\}$, where $C_{\mathrm{Pos}}$ corresponds to a bipolar vector over the set {+1,−1} indicating the state of positive edges; $C_{\mathrm{Neg}}$ refers to a bipolar vector for the transformation state of negative edges; and $C_{\mathrm{Node}}$ encodes the attributes of nodes. Given a gene $g_{i}$, if $g_{i}$ belongs to $C_{\mathrm{Pos}}$ or $C_{\mathrm{Neg}}$, a value of –1 signifies that the corresponding edge’s sign is flipped, whereas +1 implies that the original sign is retained; if $g_{i}$ is located to $C_{\mathrm{Node}}$, the value indicates the attribute assigned to the corresponding node. Figure 6 provides a visual illustration of this representation.

**Figure 7:**
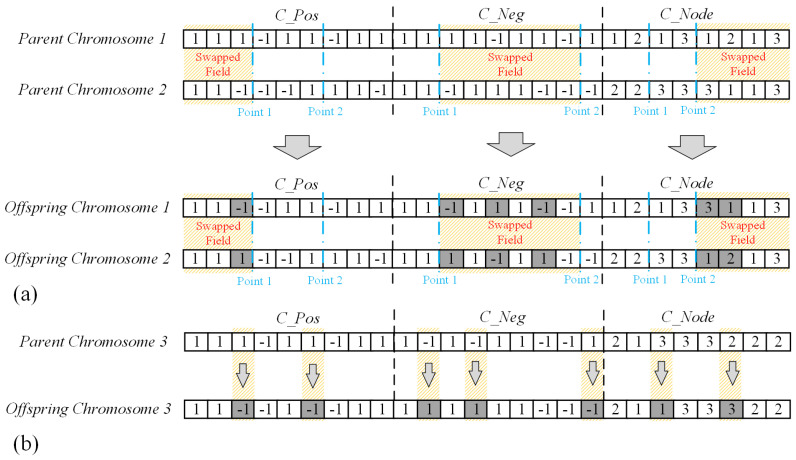
An illustration of the genetic operations: (**a**) The two-point crossover process, where the “Swapped Field” (highlighted in red fonts) indicates the gene segments exchanged between Parent Chromosome 1 and 2 at the randomly selected Point 1 and Point 2 (blue fonts). Different light-colored backgrounds are used to distinguish the origin of the segments in the resulting offspring. (**b**) The one-point mutation process, where a single gene in Parent Chromosome 3 is randomly selected and modified to produce Offspring Chromosome 3 (the mutated gene is highlighted with a gray background). In both panels, colored fonts and specific background highlighting are used to clearly indicate the genes that have been swapped or mutated from their initial states.

The Genetic-Operation(·) function consists of both crossover and mutation operations. In the crossover step, a two-point crossover strategy is adopted in this algorithm. The procedure operates as follows: two parent chromosomes, denoted as Parent Chromosome 1 and Parent Chromosome 2, are selected. Within each substring ($C_{\mathrm{Pos}}$, $C_{\mathrm{Neg}}$, and $C_{\mathrm{Node}}$) of the chromosomes, two cut points *a* and *b* are randomly chosen (a≤b≤L, where *L* is the length of the corresponding substring). A gene segment from 0 to *a*, *a* to *b*, or *b* to *L* are randomly selected. Then all the genes within the selected segment are exchanged between the two parents. An illustration of this process is provided in Figure 7a. During mutation, a chromosome is first randomly selected. A one-point mutation is then applied (as shown in Figure 7b): a gene is randomly chosen, and its value is modified based on its type. For a gene in the substring $C_{\mathrm{Node}}$, the value is replaced by other value of the node attribute. For an edge sign gene in $C_{\mathrm{Pos}}$ or $C_{\mathrm{Neg}}$, the value is flipped to its opposite sign. The number of mutations is randomly determined within the range $\left[1,N_{\mathrm{pos}}+N_{\mathrm{neg}}+N\right]$.

To mitigate the risk of premature convergence—a common pitfall in evolutionary algorithms—we incorporate a local search procedure, a technique exemplified by methods such as hill climbing [23] or simulated annealing [27]. As outlined in Algorithm 3, our local search mechanism systematically examines each gene within a chromosome to assess if a value change reduces the objective function. If a modification to either an edge sign or a node attribute yields a lower cost or improved balance, the change is accepted. This fine-grained refinement ensures that the algorithm effectively exploits local regions of the search space.
**Algorithm 3** Local Search**Require:** 
$C_{\mathrm{offspring}}$ (Offspring chromosome derived by genetic operation), *N* (Network size), NumAtt (Number of node attributes), $N_{\mathrm{pos}}$ (Number of positive edges), $N_{\mathrm{neg}}$ (Number of negative edges)  1:Create a list List with the disordered sequence from 1 to $N_{pos}+N_{neg}+N$  2:**for** i=1 to $N_{\mathrm{pos}}+N_{\mathrm{neg}}+N$ **do**      3:    $C_{\mathrm{offspringNew}}\leftarrow C_{\mathrm{offspring}}$      4:    **if** $List\left(i\right)\leq N_{\mathrm{pos}}+N_{\mathrm{neg}}$ **then**          5:        $C_{\mathrm{offspringNew}}\left(List\left(i\right)\right)\leftarrow -1\cdot C_{\mathrm{offspringNew}}\left(List\left(i\right)\right)$          6:        **if** $\mathrm{OBJ}\left(C_{\mathrm{offspringNew}}\right)>\mathrm{OBJ}\left(C_{\mathrm{offspring}}\right)$ **then**              7:              $C_{\mathrm{offspringNew}}\left(List\left(i\right)\right)\leftarrow -1\cdot C_{\mathrm{offspringNew}}\left(\mathrm{List}\left(i\right)\right)$          8:        **end if**     9:    **else**       10:        **for** c=1 to NumAtt **do**            11:              $C_{\mathrm{offspring}}\left(List\left(i\right)\right)\leftarrow c$            12:              **if** $OBJ\left(C_{\mathrm{offspring}}\right)<OBJ\left(C_{\mathrm{offspringNew}}\right)$ **then**                13:                 $C_{\mathrm{offspringNew}}\left(\mathrm{List}\left(i\right)\right)\leftarrow c$            14:            **end if**       15:        **end for**   16:   **end if**17:**end for**18:**Output:** $C_{\mathrm{offspringNew}}$

### 3.2. Complexity and Scalability Analysis

To evaluate the practical applicability of the proposed method, we analyze its computational complexity. Let *n* and *m* denote the number of nodes and edges in the network, respectively. In each iteration, the Genetic Algorithm (GA) component performs two-point crossover and one-point mutation operators for a population of size PoolSize. Given that the energy function calculation requires O(m) and each chromosome contains *n* elements, the complexity for the GA phase is O(PoolSize·(n+m)). The local search mechanism, designed to refine solutions, constitutes the primary computational cost. Considering the most *n* edge-neighbors and *n* node-neighbors for each node across *c* clusters, the local search complexity per iteration is $O(cmn^{2})$. Consequently, the total complexity of the Memetic Algorithm is $O(cmn^{2})$. Although the theoretical complexity is polynomial, the algorithm demonstrates strong scalability for large-scale social networks. This is because most real-world signed networks are sparse (where *m* is often proportional to *n*), and the local search can be further optimized by focusing on active subgraphs, making the approach feasible for networks with thousands of nodes and beyond.

## 4. Experiments

This section evaluates the performance of the proposed algorithms on a series of computer-generated and four real-world networks. All experiments are conducted on a workstation equipped with a 32-core processor and 128 GB of memory, utilizing MATLAB 24.2.0.2773142 (R2024b) (MathWorks, Natick, MA, USA) for execution. The essential parameters configurations employed in the experimental study are summarized in Table 1.

The essential parameters employed in this study are summarized in Table 1, with their values grounded in preliminary sensitivity analyses and established heuristic principles. Specifically, a larger PopSize or PoolSize enhances search precision but at the expense of computational overhead; thus, we adopted a value of 100, which provides a satisfactory balance between solution quality and time complexity [16,17,18]. The TournSize is set to 2 to moderate selection pressure, ensuring sufficient population diversity to avoid premature convergence to local optima [28]. Furthermore, the crossover ($P_{c}=0.8$) and mutation ($P_{m}\;=\;0.2$) [29] probabilities were tuned to synchronize global exploration and local exploitation. Crucially, the penalty coefficient β in Equation (3) plays a pivotal role in regulating the trade-off between the total transformation cost and the attainment of structural balance. Within the objective function, β acts as a soft constraint that enforces the energy function E(G) to converge to zero. Through a systematic series of pilot experiments, it was observed that β=1 consistently steers the network toward a fully balanced state (representing a state of perfect structural stability) while preserving the algorithm’s efficacy in minimizing modification costs. Therefore, β=1 is adopted as the standard threshold in this study to focus on the optimal dynamic transformation process.

### 4.1. Results for Computer-Generated Networks

The computer-generated networks in our study are constructed according to the benchmark networks introduced by Girvan and Newman [27]. We begin with a perfectly balanced network comprising 128 nodes partitioned into four clusters (nodes 1–32, 33–64, 65–96, 97–128). In this structure, each node has an average degree of 16, with an equal distribution of 8 links to nodes within the same cluster and 8 to nodes in different clusters. The edge signs are assigned such that all intra-cluster connections are positive, while all inter-cluster connections are negative. Regarding node attributes, identical values are assigned to nodes belonging to the same cluster, whereas distinct values are assigned across different clusters. To simulate structural imbalance, a perturbation parameter $\mu_{1}$ is introduced, which represents the fraction of edges that violate the ideal balanced state (i.e., negative edges within clusters or positive edges between clusters). By varying $\mu_{1}$, a series of networks with increasing imbalance are generated for testing.

**Figure 8:**
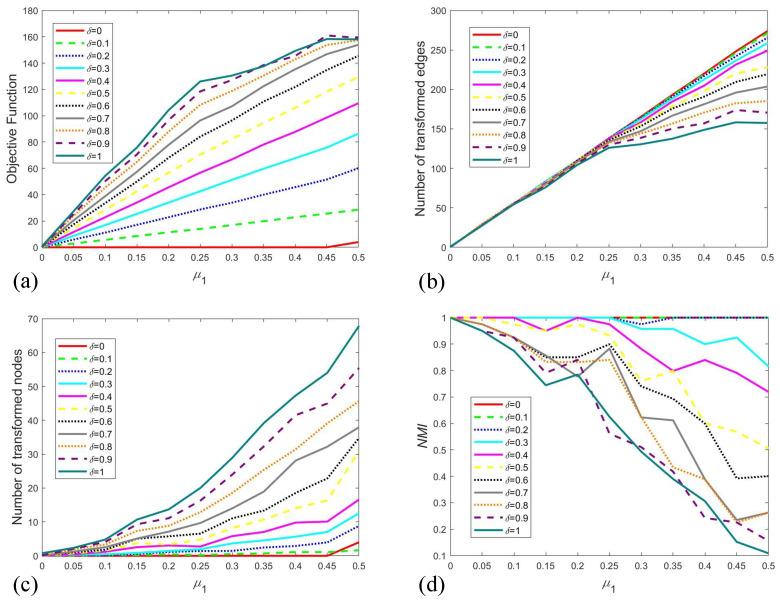
Mean value of 20-runs results for different settings of δ and $\mu_{1}$. (**a**) is the result of objective function, (**b**) is the result of the number of transformed edges, (**c**) is the number of transformed nodes, (**d**) is the NMI result.

Figure 8 illustrates the averaged outcomes from 20 independent runs across various settings of δ and $\mu_{1}$. As shown in Figure 8a, the objective function value increases with $\mu_{1}$, reflecting the escalating computational and structural costs required to restore balance in heavily perturbed networks. This trend demonstrates the sensitivity of the optimization process to structural perturbations. Concurrently, a larger δ also leads to an elevation in the objective function. Since δ represents the unit cost of modifying an edge, a higher value directly translates to a greater total expense for adjustments, thereby inflating the objective function. The number of transformed edges, presented in Figure 8b, grows as $\mu_{1}$ increases. However, this growth rate is attenuated with rising δ, because the transformation process is partially supplanted by the transformation of node attributes—a cheaper alternative under high edge-adjustment costs. From Figure 8c, we can find the number of transformed nodes exhibits a positive correlation with both δ and $\mu_{1}$. These observations collectively confirm the adaptability of our method to different scenarios by tuning the parameter δ.

To quantify the consistency between the initial attributes and the optimized results, we utilize the Normalized Mutual Information (NMI) index. For two attribute assignments *A* and *B* over *n* nodes, a confusion matrix $C=\|C_{ij}\|$ is constructed, where the entry $C_{ij}$ denotes the number of nodes belonging to attribute *i* in *A* and attribute *j* in *B*. NMI between *A* and *B* is then calculated as follows:

(4) $$NMI\left(A,B\right)=\frac{-2\sum_{i=1}^{C_{A}}\sum_{j=1}^{C_{B}}C_{ij}\log\left(\frac{C_{ij}n}{C_{i.}C_{.j}}\right)}{\sum_{i=1}^{C_{A}}C_{i.}\log\left(\frac{C_{i.}}{n}\right)+\sum_{j=1}^{C_{B}}C_{.j}\log\left(\frac{C_{.j}}{n}\right)}$$

where $C_{A}$ and $C_{B}$ represent the number of attribute kinds in *A* and *B*, $C_{i.}$ and $C_{.j}$ denote the sum of elements in the $i^{th}$ row and $j^{th}$ column of the confusion matrix *C*. The value of NMI ranges from 0 to 1, with a higher value indicating a greater degree of consistency between the two assignments. The NMI results under different δ and $\mu_{1}$ settings are displayed in Figure 8d. Our analysis reveals that NMI remains at 1 as $\mu_{1}$ increases when δ is small. In contrast, a declining trend in NMI is observed with rising $\mu_{1}$ under larger δ values. This pattern underscores our algorithm’s capability to effectively navigate the trade-off between structural modification and the incurred cost.

**Figure 9:**
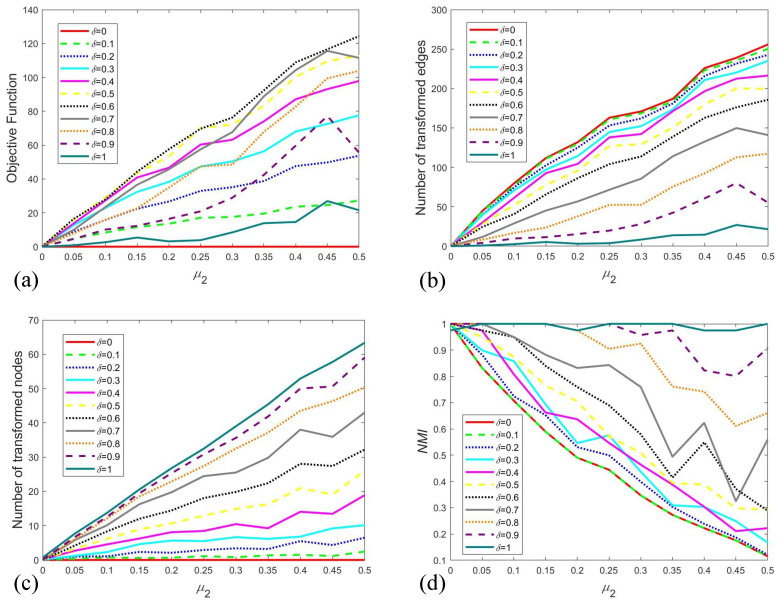
Mean value of 20-runs results for different settings of δ and $\mu_{2}$. (**a**) is the result of objective function, (**b**) is the result of the number of transformed edges, (**c**) is the number of transformed nodes, (**d**) is the NMI result.

Subsequently, we investigate imbalance induced by node attributes. A parameter $\mu_{2}$ is introduced to quantify the level of node-attributed imbalance, defined as the proportion of nodes within a cluster that possess divergent attributes. This disturbance is implemented through a random process, where each cluster has an identical probability $\mu_{2}$ of containing nodes with heterogeneous attributes. Figure 9 presents the averaged results for various δ and $\mu_{2}$. Similar to the previous findings, the objective function increases with $\mu_{2}$. However, it exhibits an inverted U-shaped pattern in response to δ. This non-monotonic behavior arises from a strategic shift. When δ is smaller, the system preferentially modifies edges to attain structural balance, and a larger δ causes a higher total cost. In contrast, larger δ makes edge adjustments prohibitively expensive, causing the model to increasingly flip node attributes instead, which in turn reduces the total cost. Consequently, as shown in Figure 9b, the number of transformed edges grows with $\mu_{2}$, but this growth is progressively suppressed as δ increases. Consistent with this reasoning, Figure 9c demonstrates that the number of transformed nodes rises with both δ and $\mu_{2}$. The NMI result in Figure 9d reveals a contrasting trend to Figure 8d. Here, NMI remains around 1 for large δ as $\mu_{2}$ increases, whereas it declines for small δ. This observation confirms that our algorithm is effective not only for resolving conflicts arising from relational imbalance but also for mitigating those caused by inconsistencies in node attributes.

**Figure 10:**
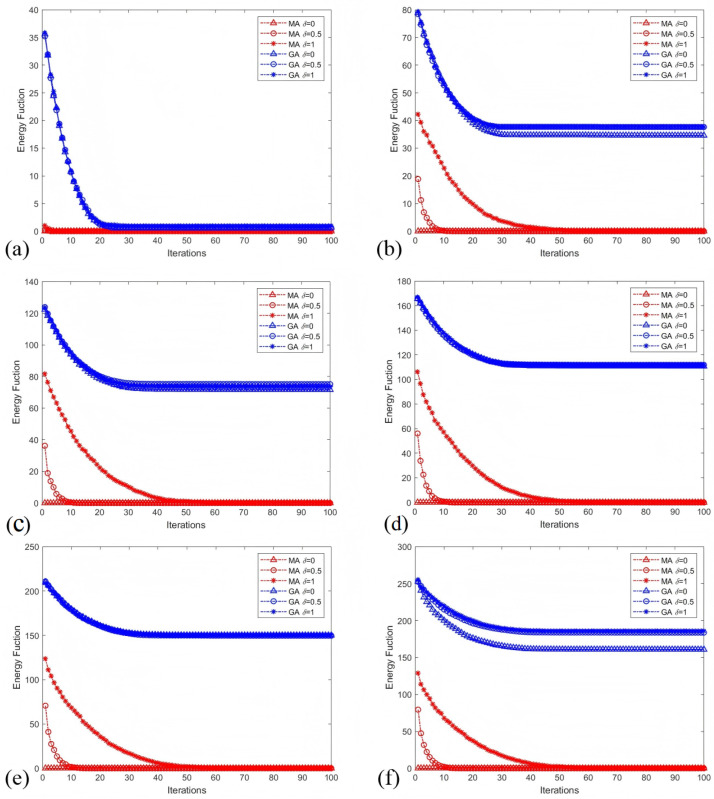
Mean value of 20-runs results of the energy function for each iteration acquired by MA and GA with different δ and $\mu_{1}$. (**a**–**f**) are, respectively, the results of $\mu_{1}=0,0.1,0.2,0.3,0.4,0.5$.

Finally, we performed a comparative analysis between the proposed Memetic Algorithm (MA) and a standard Genetic Algorithm (GA). Convergence behavior was evaluated by tracking the energy function across iterations on networks with varying $\mu_{1}$. Figure 10 illustrates that MA achieves a significantly accelerated convergence rate and reaches a final solution with markedly lower residual energy compared to the GA. This advantage underscores the efficacy of the synergistic coupling between global genetic exploration and local heuristic refinement in driving the network toward a globally balanced state.

### 4.2. Results for Real-World Networks

The proposed method is also evaluated on four real-world networks over 20 independent runs:

*Slovene Parliamentary Party Network (SPP)*: A relational network of 10 political parties, established by parliamentary experts in 1994 [30].

*Illustrative Signed Network (ISN)*: A network with 28 nodes and 42 edges, originally presented by Yang et al. [31].

*Epidermal Growth Network (EGN)*: The network representing the Epidermal growth factor receptor pathway [32], containing 330 nodes and 852 edges.

*Macrophage Network (MN)*: A molecular interaction map for a macrophage [33], comprising 678 nodes, 947 positive edges, and 478 negative edges.

All networks are connected. Due to the scarcity of publicly available datasets that integrate both edge signs and node attributes, we followed the methodology of Sun et al. [34] and Du et al. [18] by manually or randomly assigning attributes to nodes. *SPP* is nearly balanced, partitionable into two opposing communities with only two intra-cluster negative edges causing imbalance. *ISN* is perfectly balanced, divisible into two hostile clusters. For *SPP* and *ISN*, node attributes were assigned via two protocols: (1) random binary assignment, and (2) manual assignment based on domain knowledge. For the larger *EGN* and *MN* networks, a random 4-category attribute was assigned to each node, consistent with prior work [17].

The results for different δ values are summarized in Table 2. Notably, the energy function consistently converges to zero across all parameter settings, confirming the algorithm’s effectiveness in achieving structural balance. As δ increases, the objective function displays an inverted U-shaped pattern, while the number of transformed edges declines and the count of transformed nodes rises. This trend demonstrates the algorithm’s strategic adaptability: it shifts the optimization burden from edge modifications to node attribute changes as the relative cost of edge operations (δ) becomes prohibitive.

**Figure 11:**
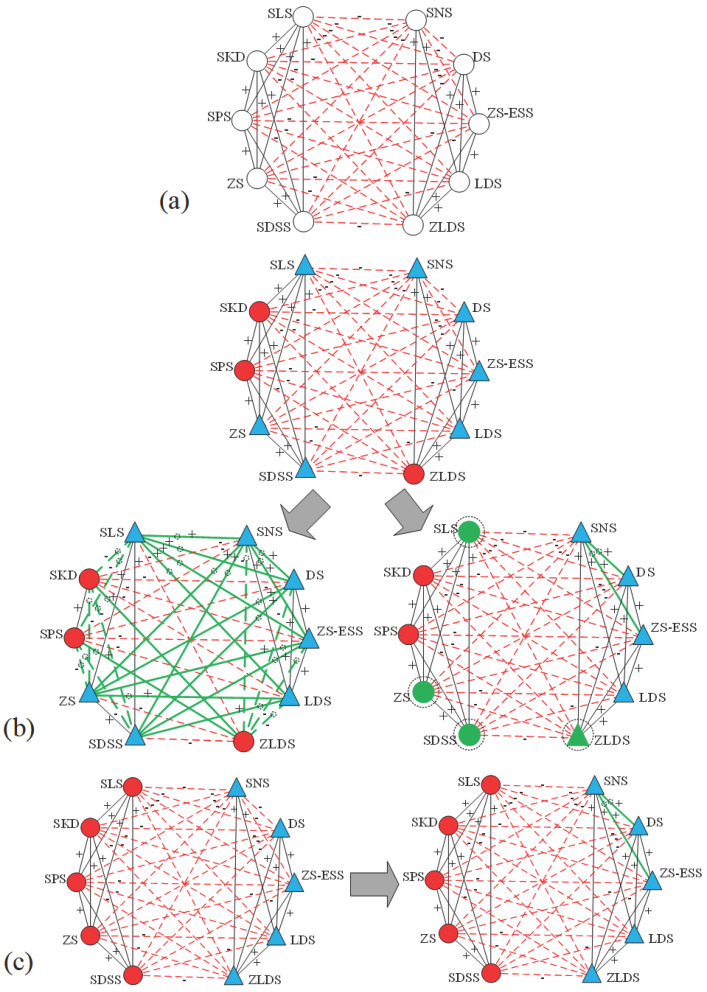
Transformation strategy of the *SPP* network with different node attribute assignments and varying δ. (**a**) The original network without node attributes; the upper part of (**b**) is the network with random node attributes, the lower left shows the transformation strategy for δ≤0.5, and the lower right shows the strategy for δ>0.5; the left part of (**c**) is the network with documented node attributes, and the right part shows the transformation strategy applicable to all values of δ. Edges marked with “+” indicate positive relationships (solid lines), while those with “−” indicate negative relationships (dashed lines). Node attributes are distinguished by varying colors and shapes (e.g., red circles and blue triangles, consistent with Figure 4). Bold green lines and green-colored nodes (e.g., green circles or triangles) highlight the edges and nodes that have undergone transformation, respectively.

**Figure 12:**
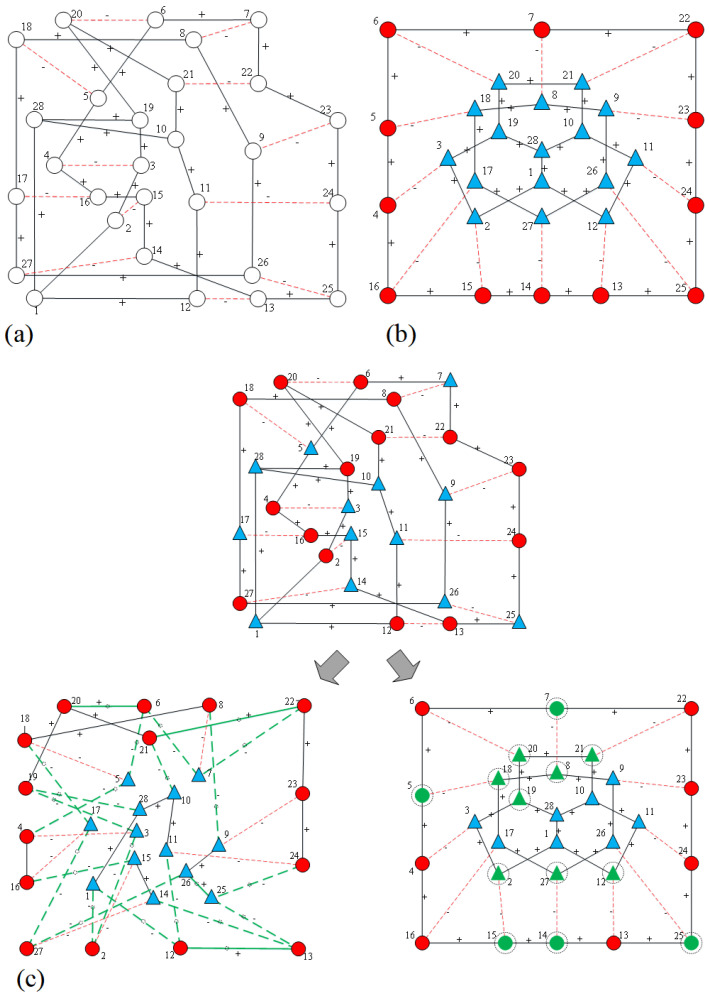
Transformation strategy of the *ISN* network with different node attribute assignments and varying δ. (**a**) The original network without node attributes; (**b**) the network with documented node attributes, which is already balanced; the upper part of (**c**) is the network with random node attributes, where the lower left shows the strategy for δ≤0.5, and the lower right shows the strategy for δ>0.5. Edges marked with “+” indicate positive relationships (solid lines), while those with “−” indicate negative relationships (dashed lines). Node attributes are distinguished by varying colors and shapes (consistent with Figure 11). The dashed circular lines denote the boundaries of the balanced communities formed after the transformation (consistent with Figure 6). Bold green lines and green-colored nodes (e.g., green circles or triangles) highlight the specific edges and nodes that have undergone transformation, respectively.

In the proposed method, every solution is decoded into a specific transformation strategy. The resulting structural adjustments for the *SPP* and *ISN* networks are visualized in Figure 11 and Figure 12, respectively. Figure 11a depicts the original *SPP* network without assigned node attributes, while the upper part of Figure 11b shows the network after the random assignment of attributes. The results indicate that at lower δ values (δ≤0.5), the algorithm prioritizes flipping the signs of unbalanced edges over altering node attributes, forming two distinct communities. In contrast, a higher δ value triggers a different paradigm: the attributes of four nodes are modified, accompanied by the conversion of two negative edges into positive ones. In the experiment with documented attributes (Figure 11c), only the two unbalanced negative edges are reversed. This outcome aligns with Wang et al. [12], confirming that our algorithm is backward-compatible with conventional signed networks that do not incorporate node attributes.

Figure 12a displays the original *ISN* without assigned node attributes, while Figure 12c shows the network after nodes are randomly assigned certain properties. Similar to the result observed in Figure 11b, when δ≤0.5, the signs of unbalanced edges are flipped without modifying node attributes. In contrast, when δ exceeds 0.5, attributes of 13 nodes are transformed, while no edge signs are altered. This occurs because *ISN* is inherently relationally balanced when not considering node attributes. If node attributes are subjectively assigned based on prior knowledge, as illustrated in Figure 12b, the resulting fully signed *ISN* adheres to both edge and node balance principles [17]. Under such conditions, structural balance is achieved without modifying any edges or nodes. By tuning the parameter δ, the desired outcome can be precisely obtained.

## 5. Conclusions

### 5.1. Summary and Theoretical Contributions

Structural balance is a fundamental tenet in signed social network analysis. This paper proposes a unified optimization framework for the dynamic transformation of structural balance in fully signed networks, reformulating the evolutionary transition as a constrained optimization problem. To solve this, a memetic-based algorithm was developed to minimize a cost-sensitive objective function. Extensive evaluations on both synthetic and real-world networks demonstrate how the objective function responds to varying transformation costs, validating the effectiveness of the proposed algorithm in steering structural balance dynamics.

This work offers a robust framework for analyzing network configurations that simultaneously incorporate edge signs and node attributes, thereby extending the methodological repertoire of social computing. In social network analysis, it is common to examine the interplay between relational patterns and demographic variables. We identify three distinct aggregation modes grounded in the basic network components—nodes, edges, and structure: (1) homophily-driven nodal aggregation, where attribute similarity leads to macroscopic segregation [19]; (2) balance-theory-based edge aggregation, which partitions populations into mutually opposing factions [10]; and (3) structural aggregation, manifested as community or cohesive subgroup formation characterized by dense intra-group ties [35]. By integrating these three modes, our model provides a holistic perspective to explore the linkage between micro-level individual behaviors and macro-level collective phenomena.

From the perspective of Computational Social Science, this integrated model serves as a quantitative tool to investigate social dynamics such as group polarization and segregation. It offers strategic insights into how social systems reach equilibrium through the synchronous adjustment of “who individuals are” (attributes) and “how they interact” (relationships), providing a more nuanced representation of genuine human dynamics.

### 5.2. Limitations and Future Work

Despite the promising results, this study has certain limitations regarding the interpretability of real-world cases. Due to the current scarcity of datasets containing both relational signs and authentic node attributes, some attributes in our experiments were assigned randomly, while this approach effectively verifies the algorithm’s computational efficiency and convergence, it may constrain the depth of sociological interpretation in those specific instances.

However, the primary contribution of this work remains the development of a generalized and extensible optimization framework. As more comprehensive datasets with verified individual attributes emerge, this model can be readily deployed to uncover deeper social insights. Furthermore, while the current study employs a penalty coefficient to balance costs and structural integrity, the transition to multi-objective optimization represents a promising future trajectory. Simultaneously optimizing multiple competing objectives—such as minimizing modification costs while maximizing group cohesion or stability—would provide a diverse set of Pareto-optimal solutions to support complex social decision-making. Future research will focus on applying this framework to empirical datasets with verified attributes and exploring these multi-objective evolutionary strategies to further enhance practical interpretability and algorithmic flexibility.

## Figures and Tables

**Table 1:** Essential parameters employed in the experimental study.

Parameter	Meaning	Value
PopSize	Population size	100
TournSize	Tournament size	2
PoolSize	Mating pool size	100
$P_{c}$	Crossover probability	0.8
$P_{m}$	Mutation probability	0.2
β	Penalty coefficient	1

**Table 2:** Mean value of 20-runs results on four real-world networks.

Networks	Index	δ = 0	δ = 0.1	δ = 0.2	δ = 0.3	δ = 0.4	δ = 0.5	δ = 0.6	δ = 0.7	δ = 0.8	δ = 0.9	δ = 1
*SPP*–random attribute	Objective function	1.25	3.88	6.47	7.81	9.27	11.30	12.77	14.94	12.68	11.03	8.05
	Transformed edges	22.05	21.70	22.55	22.65	20.25	20.50	19.85	20.55	15	11.85	8.05
	Transformed nodes	1.25	1.90	2.45	1.45	1.95	2.10	2.15	1.85	3.40	3.65	3.35
	Energy Function	0	0	0	0	0	0	0	0	0	0	0
*SPP*–documented attribute	Objective function	0	0.20	0.40	0.60	0.80	1.00	1.20	1.40	1.60	1.80	2.00
	Transformed edges	2.00	2.00	2.00	2.00	2.00	2.00	2.00	2.00	2.00	2.00	2.00
	Transformed nodes	0	0	0	0	0	0	0	0	0	0	0
	Energy Function	0	0	0	0	0	0	0	0	0	0	0
*ISN*–random attribute	Objective function	4.75	5.29	7.04	8.66	11.99	12.10	14.34	14.15	13.52	14.20	14.80
	Transformed edges	21.65	22.30	22.00	20.70	20.15	18.55	18.30	16.40	14.90	14.60	14.80
	Transformed nodes	4.75	3.40	3.30	3.50	6.55	5.65	8.40	8.90	8	10.60	10.00
	Energy Function	0	0	0	0	0	0	0	0	0	0	0
*ISN*–documented attribute	Objective function	0	0	0	0	0	0	0	0	0	0	0
	Transformed edges	0	0	0	0	0	0	0	0	0	0	0
	Transformed nodes	0	0	0	0	0	0	0	0	0	0	0
	Energy Function	0	0	0	0	0	0	0	0	0	0	0
*EGN*–random attribute	Objective function	124.65	178.03	218.28	249.39	270.91	296.95	321.32	339.75	340.66	349.50	348.45
	Transformed edges	449.15	448.25	440.80	431.60	424.75	412.05	406.40	397.90	372.35	365	348.45
	Transformed nodes	124.65	148	162.65	171.30	168.35	181.85	193.70	204.05	213.90	210	233.95
	Energy Function	0	0	0	0	0	0	0	0	0	0	0
*MN*–random attribute	Objective function	314.80	324.50	419.09	484.63	510.35	580.40	604.33	634.77	638.52	624.82	622.45
	Transformed edges	821.55	818.60	807.25	793.75	765.65	753.80	729.75	715.20	682.25	644.85	622.45
	Transformed nodes	314.80	269.60	322.05	352.15	340.15	407.00	416.20	447.10	463.60	444.55	491.25
	Energy Function	0	0	0	0	0	0	0	0	0	0	0

## Data Availability

The data presented in this study are available on request from the corresponding author. The data are not publicly available due to privacy restrictions.

## Abbreviations

The following abbreviations are used in this manuscript:

NP-hard	Nondeterministic Polynomial-time hard
MA	Memetic Algorithm
NMI	Normalized Mutual Information
